# Blood lines

**DOI:** 10.1038/sj.bjc.6602727

**Published:** 2005-08-16

**Authors:** T R J Lappin

**Affiliations:** 1 Belfast City Hospital Department of Haematology, Centre for Cancer Research and Cell Biology, Queen's University, University Floor, Tower Block, Lisburn Road, Belfast BT9 7AB, N Ireland

Written in a clear and persuasive style, Donald Metcalf's *Blood Lines* is a highly readable and instructive guidebook for conducting haematological analysis of a novel mouse. Its subtitle, *An Introduction to Characterizing Blood Diseases of the Post-Genomic Mouse*, alludes to the practicalities of producing designer mice utilising the current detailed knowledge of the murine genome.

Knockout mice are created to examine the function of a preselected gene. In practice, a mutant form of the target gene is constructed in the laboratory and swapped with the functional copy of the gene in mouse embryonic stem cells. The resulting phenotype may provide some clues about the role of the mutant gene in the mouse. Since nature is parsimonious, knockout mouse models have been widely adopted to gain insights into gene function in human diseases.

Phenotypic characterisation of a genetically modified mouse is not a trivial exercise. Metcalf empathises with the newcomer to the field who has the daunting task of characterising the disease in a novel mouse that has become prematurely ill. He lays the foundations by describing a comprehensive method for identifying subtle phenotypic variations. He stresses the need for a detailed systematic analysis, which involves examining animals of different ages and stages of development, always comparing them with appropriate control animals. For the haematopoietic system, this involves examination of progenitor cells not only in the blood and marrow but also in the spleen and liver, which recapitulate embryonic blood cell production during stress haematopoiesis in the adult.

The principal feature of haematopoietic culture is the heterogeneity of the proliferating clones. As would be expected from a pioneer of culture studies *in vitro*, Metcalf shows that important information can be gleaned from detailed analysis of the clonal response to different haematopoietic growth factors over a range of concentrations. This is exemplified by his investigation of the SOCS-3 knockout mouse. Marrow cells showed superficially normal responses to G-CSF or IL-6, as assessed by titration curves. Unexpectedly, further analysis using differential colony counts revealed that SOCS-3^−/−^ cells formed macrophage-containing colonies of quite large size as the concentrations of IL-6 or G-CSF were *reduced*. This contrasted sharply with the highly granulocytic nature of colonies of normal cells found in response to either growth factor. When injected with G-CSF, the marrow of SOCS-3^−/−^ mice became packed with actively proliferating granulocytic cells, which invaded adjacent muscle tissue, the spinal canal and the liver. Taken together, these observations provided valuable evidence that SOCS-3 is a critical negative physiological regulator of G-CSF signalling and emergency granulopoiesis.

Throughout the book, the reader is struck by the author's clarity of rationale for undertaking the laboratory investigations and his scientific rigour. Metcalf advocates presenting data as actual colony counts and condemns manipulation of data such that the absolute data disappear. Attesting to the author's meticulous attention to detail, the gender status of experimental animals is verified by differences in the salivary gland, Bowman's capsule in the kidney and liver nuclei.

The scrupulous detail of Metcalf's lucid text is mirrored by the excellent reproduction of colour figures, a crisply printed format, and a binding that enables the book to lie flat when opened. This feature will be welcomed by the bench scientist using the book as a laboratory manual.

Donald Metcalf is clearly at ease with his subject and demonstrates an enthusiasm for laboratory work, which appears undiminished after more than 50 years. His book is a *tour de force* of experimental investigation, and provides essential insights for anyone contemplating work on transgenic mouse models. It will be a valuable addition for anyone interested in elucidating gene function in human disease.

